# Investigation of Praseodymium Ions Dopant on 9/65/35 PLZT Ceramics’ Behaviors, Prepared by the Gel-Combustion Route

**DOI:** 10.3390/ma16237498

**Published:** 2023-12-04

**Authors:** Małgorzata Płońska, Julian Plewa

**Affiliations:** Faculty of Science and Technology, Institute of Materials Engineering, University of Silesia in Katowice, 75 Pułku Piechoty Street 1a, 41-500 Chorzów, Poland; julian.plewa@us.edu.pl

**Keywords:** PLZT, Pr^3+^-dopant, gel-combustion synthesis, dielectric materials, luminescent ceramics

## Abstract

In this work, were synthesized (Pb_0.91_La_0.09_)(Zr_0.65_Ti_0.35_)_0.9775_O_3_ ceramic materials with different concentrations of praseodymium (0, 0.1, 0.3, 0.5, 1 wt.%) via gel-combustion route and sintered by the hot uniaxial pressing method. Measurements were conducted on the obtained ceramics using X-ray powder diffraction (XRD), scanning electron microscope (SEM), EDS analysis, and examination of dielectric and ferroelectric optical properties. Results give us a detailed account of the influences of the praseodymium ions on the structural, microstructural, and dielectric properties. 3D fluorescence maps and excitation and emission spectra measurements show how a small admixture changes the ferroelectric relaxor behavior to an optically active ferroelectric luminophore.

## 1. Introduction

Perovskite ceramics ABO_3_ are continuously interesting engineering materials, extensively commercialized and used in various devices, such as sensors and actuators. The complex oxide compound lead-lanthanum zirconium titanate (PLZT) is particularly important among them [1].

PLZT ceramics represent multifunctional and intelligent material, well-known for its comprehensive applications as resonators, ultrasonic transducers, thermistors, or capacitors [2]. These high ferroelectric, piezo-, and dielectric properties are consequences of non-stoichiometry in the cationic subnetwork in the PLZT atomic structure. Therefore, structural defect B-subnetwork within its perovskite structure is described and taken into account in the material’s chemical formula [3,4] as follows:

(Pb_1−*x*_La*_x_*)(Zr*_y_*Ti_1−*y*_)_1−0.25*x*_V^B^_0.25*x*_O_3_ (1)

where V^B^ means a defect in subgrid B, hence the notation 100(*x*/*y*/1 − *y*) PLZT.

As is widely known, extensive research has been conducted in doped PLZT systems to enhance the performance of this material. Although PLZT ceramics have been studied over the past decades, there is no systematic study of this material for use as a luminophore [5]. The doping with rare earth metals of oxide compounds with perovskite-type structures ABO_3_ usually changes their dielectric and ferroelectric properties. Of course, several publications, i.e., [6,7,8,9], present this issue, but it still requires supplementation, especially the confirmation of the obtained results. In most cases, when doing so, a photoluminescence effect has also been achieved [10,11,12]. Interestingly, structural defects are the reason why pure PLZT exhibits poor luminescence properties (344 µm excitation, 500 nm emission) [13]. On the other hand, they do not prevent and even favor the introduction of additional lanthanide ions without any restriction up to relatively high concentrations [14]. Consequently, the electro-optical effects of such doped ceramics are up to several times higher than in other optically active crystals, making PLZT:RE^3+^ ceramics an alternative material in the development of solid-state lasers, e.g., for near-infrared applications [15]. This is probably the reason why most researchers focus only on the optical behavior of PLZT:RE^3+^, i.e., [16,17,18] without studying the effect of doping on other properties. The authors do not provide information on how the ferroelectric state or other dielectric parameters of such materials change, focusing mainly on describing spectral phenomena without looking for correlation with the ferroelectric ceramic matrix features, i.e., [19,20,21].

**Figure 1:**
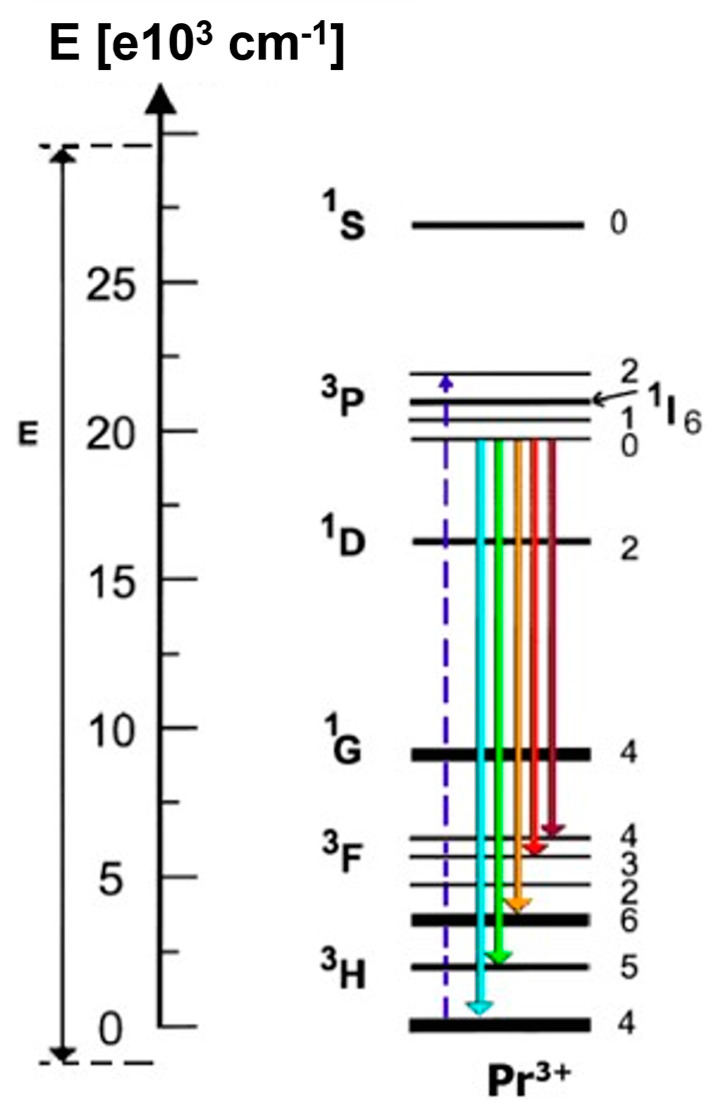
Simplified energy level diagram of Pr^3+^ in PLZT ceramics.

Since the (Pb_0.91_La_0.09_)(Zr_0.65_Ti_0.35_)_0.9975_O_3_ transparent ceramics show excellent optical quality and ferroelectric properties due to their relaxor-like properties, they have become interesting for applications in many electro-optical devices [22,23,24,25,26,27,28]. By doping with rare earth metals, 9/65/35 PLZT ceramics can become a multifunctional (smart) material [29,30,31]. This solid solution system, with bright ecru colors, can be an attractive ceramic matrix (host material), for example, for praseodymium ions (Pr^3+^); consequently, it can be expected that not only dielectric and ferroelectric properties but also luminescent properties will be obtained [21,32]. From the studies of various authors, it appears that the magnitude of the energy gap *E_g_* for PLZT is about 3 eV. However, this magnitude strongly depends on the concentration of La^3+^ ions [33,34]. The electron transitions of Pr^3+^ ions fall within this range according to the Dieke diagram (Figure 1) [35].

Doping with Pr^3+^ ions will provide a red emission signal, and it is expected that this process may also enhance the dielectric and ferroelectric properties [36]. The dielectric properties of PLZT doped with rare earth metals have been improved many times [30,31,37]. In emission, it can be induced by excitation with both UV and blue light. It has also been proved that titanates–zirconates of alkaline earth metals naturally tend to form defects [38] and the vacancies are formed during the sintering of PLZT ceramics. These vacancies result in blue emission (420–480 nm) and dark red emission (718 nm) upon UV excitation [39], which is more pronounced at low temperatures because the emission of defect structures is higher. Although the author previously described the behavior of 8/65/35 PLZT doped with praseodymium ions using the conventional mixed oxide method (MOM) [40], the influence of Pr^3+^ on the properties of 9/65/35 PLZT by has not been previously described. All these facts motivated us to carry out this study.

Techniques for producing co-doped polycrystalline PLZT ceramics have evolved since [41]. There is a gradual departure from the conventional solid-state reaction between the constituent oxides. A high-temperature process (~950 °C during synthesis to ~1250 °C into sintering), carried out for a prolonged time, is necessary to gain the pure phase, resulting in significant grain growth [40,42]. In this process, extensive milling of the powders can lead to their possible contamination, inhomogeneous or non-stoichiometry. As a result, it often reduces the properties of ceramic products.

One alternative method to the MOM technique for obtaining powders is the low-temperature gel-combustion synthesis method. This low-cost technique is well known for synthesizing numerous materials and fabricating several ceramic powders [43]. This method is classified as the so-called wet chemical method because it is first realized in an aqueous solution [44]. The oxide powders blend well with inorganically derived metal salts (such as nitrates and sulfates). Ingredients are dissolved in a solution containing mixed combustion agents (i.e., citrate acid, polyacrylic acid, trisamine, or urea), which can act as the metal ion complexing agent and fuel the synthesis of oxide powders [45].

In summary, the gel-combustion technique is easy, time-saving, and energy-efficient for synthesizing very fine powders. This self-propagating synthesis method can directly yield the final powders, or calcination of the reaction product is required [43]. Thus, it is a good method for obtaining single-phase PLZT powders doped with pure and rare earth ions.

In this work, (Pb_0_._91_La_0.09_)(Zr_0.65_Ti_0.35_)_0.9775_O_3_ undoped and praseodymium-doped powders (with 0, 0.1, 0.3, 0.5, 1 wt.%) were prepared using the self-propagating gel-combustion synthesis method. The consolidation of ceramic samples was performed by the hot uniaxial pressing method. The praseodymium dopant’s influence on the structure and optical, dielectric, and ferroelectric behaviors was studied for all samples with a composition of 9/65/35 PLZT:Pr^3+^.

## 2. Materials and Methods

Based on the chemical Formula (1), five compositions of PLZT were synthesized using Pb(NO_3_)_2_ (POCH, 99.99%), La(NO_3_)_3_·6 H_2_O (POCH, 99.99%), Zr(OCH_2_CH_2_CH_3_)_4_ (Sigma Aldrich, St. Louis, MO, USA, 70%), Ti(OCH_2_CH_2_CH_3_)_4_ (Sigma Aldrich, St. Louis, MO, USA, 97%), Pr(NO_3_)_3_·6 H_2_O (Sigma Aldrich, St. Louis, MO, USA, 99.9%), HNO_3_ (POCH, 65%), CH_3_CH_2_CH_2_OH (POCH, 99.99%), Trisamine (NH_2_C(CH_2_OH)_3_-Sigma Aldrich, St. Louis, MO, USA, 99.8%), n-propanol, and deionized water as starting materials.

**Figure 2:**
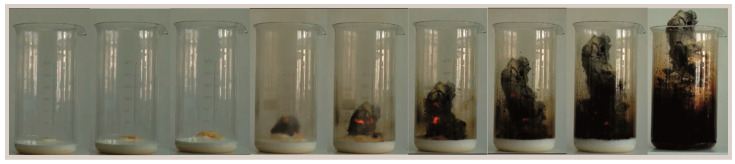
An example of the low-temperature synthesis of 9/65/35 PLZT:Pr^3+^ powders by gel-combustion method.

First, the raw materials were weighed to the nearest ±0.001 g in stoichiometric quantities. All compositions were prepared with a 5 wt.% excess of lead compound to protect them from evaporation during high-temperature processing. For each composition (Table 1), the aqueous solutions containing Pb^2+^ and La^3+^, as well as Pr^3+^ nitrates and trisamine, were prepared respectively. Similarly, to obtain the corresponding nitrates, proportional amounts of nitric acid were added to solutions of Zr^4+^ and Ti^4+^ propoxides. The prepared precursor solutions were mixed in the appropriate order with magnetic stirring. Subsequently, Trisamine was added to the solution as a reaction fuel and stabilizing agent (in a 1:2 mole ratio for each metal ion). Then, the resulting transparent sol solution was heated up to 70–80 °C and evaporated until a viscous ecru color gel was obtained. The outcome gel was heated further until the initiation of the combustion reaction. Gradual foaming of the solution and release of gases (N_2_, CO_2_) due to the decomposition of trisamine were observed. The initiation of the combustion synthesis process occurred at a point-like location, and the self-propagating combustion reaction followed a moving wave across the volume of the solution. Its duration was several seconds, and the extinction occurred spontaneously. The final result was an ashen gel with the consistency of a fine, fluffy foam (Figure 2), which contained the inorganic part and carbon residues of the combustion process.

**Figure 3:**
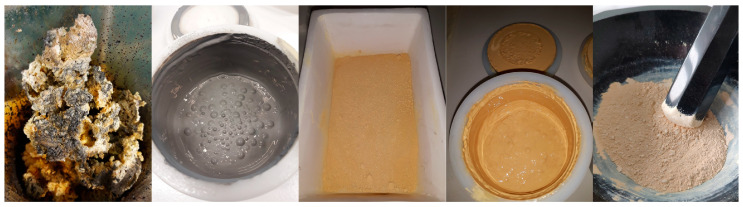
Illustration of the preparation of the 9/65/35 PLZT:Pr^3+^ powder, obtained via gel-combustion synthesis and calcined at *T* = 600 °C/4 h.

Finally, the product was mixed and ground in absolute ethyl alcohol for 24 h using a planetary ball mill, polyamide cup, and YTZ cylinders as grinding media (ZrO_2_-Y_2_O_3_, Ø = 2 and 5 mm in diameter). The slurry was then transferred to evaporate to a porcelain basin and dried in an air atmosphere for approximately 48 h. Initially, the powder was crushed and calcined in the air at *T* = 600 °C for 4 h to remove the residual organic materials. Subsequently, a yellowish ceramic powder was obtained. To eliminate agglomerates in powders, they were remilled for 24 h with similar conditions, and then it was cold-pressed into pellets in a stainless-steel die of d = 10 mm in diameter and pressed into pellets under pressure (*p* = 600 MPa). Figure 3 illustrates the sample preparation of gel-combustion synthesized powders after calcination.

The creation of vacancies or defects is a common, occurring effect in the fabrication of perovskite-type ferroelectric materials. During the sintering process, which involves high temperatures, the evaporation of volatile metallic elements leads to the formation of cation vacancies within the crystal structure. These vacancies often result in the creation of holes as a compensatory mechanism. Simultaneously, at elevated sintering temperatures, there is a loss of oxygen and the emergence of oxygen defects (oxygen vacancies). This occurs due to a charge imbalance, generating electrons through ionizing these oxygen vacancies [46].

The process of vacancy ionization resulting in the production of electrons and holes can be effectively described for PLZT using the Kröger–Vink notation [47]:

V_*Pb, La*_ ↔ V’_*Pb, La*_ + *h*· (2)

V*_O_* ↔ *V’_O_* = *e*’ or *V’_O_* ↔ *V”_O_* + *e*′ (3)

The PLZT doped with praseodymium ions Pr^3^ and oxygen vacancies might be represented as follows:

(PbLa)(ZrTi)O₃ − x Pr^3^⁺ + x/2 V*_O_*·, (4)

where x represents the concentration of oxygen vacancies compensated by praseodymium dopants.

According to the literature [48], minimizing defects in this ceramic material category can be accomplished by applying suitable technological processes. These processes encompass the incorporation of specific admixtures, conducting sintering in a controlled environment and employing high-pressure sintering techniques, among others [49]. For all these reasons, the sintering process of ceramics was carried out by hot-uniaxial pressing (HUP) at *T* = 1100 °C with a uniaxial pressure of 20 MPa for 3 h. Fabricated ceramic samples were then cut to give parallel plates of 1 mm and polished with the diamond paste to a smooth surface finish. The bulk density of the prepared samples was measured using Archimedes’s method.

Differential thermal analysis (DTA) and thermogravimetric analysis (TG) have been used to determine the thermochemical properties of obtained powders. Thermal analysis of the produced material was carried out using the MOM q1500D derivatograph (Budapest, Hungary), Paulik–Paulik–Erdey system. Transmission electron microscopy (TEM, FEI Tecnai Osiris 200 kV; Hillsboro, OR, USA) was used to determine the morphology of powders.

The phase composition of all ceramic materials was identified by X-ray diffraction (XRD; Philips PW 3710, Almelo, The Netherlands). XRD data were collected using CuKα radiation at room temperature, over a 2° range of 10–60°, with a step of 0.02° and a counting time of 1.0 s/step. Full pattern identification was made by using the X’Pert HighScorePlus software Version 5.2.0 package (created by Malvern Panalytical B.V., Almelo, The Netherlands). Data from the PDF database (International Centre for Diffraction Data (ICDD^®^)) [50] were used as a reference for the structural analysis of glass material.

The fractured surfaces of prearranged ceramics were coated with graphite for scanning electron microscopy studies (SEM, HITACHI S-4700, Tokyo, Japan). The NORAN Vantage system of microanalysis was used to qualify the chemical composition of samples.

3D fluorescence maps and excitation and emission spectra measurements were recorded on Shimadzu fluorescent spectrometer RF-6000 (Tokyo, Japan) equipped with a 150W Xenon discharge lamp in the 200–900 nm region, respectively. All spectral measurements were carried out with a resolution of 0.1 nm and at room temperature. Luminescence lifetimes were determined with an accuracy of 1 ms.

Before electrical measurements, the silver electrodes were applied to both surfaces of the annealed bulk ceramic samples and then heated in an oven (*T* = 850 °C/0.5 h) to evaporate the organic solvent from the silver paste. Temperature dielectric measurements were performed on an LCR meter (QuadTech 1920 Precision LCR Meter, QuadTech, Inc., Maynard, MA, USA). Ferroelectric properties were carried out using a virtual Sawyer-Tower circuit and a high-voltage amplifier (Matsusada Inc. HEOPS-5B6 precision, Matsusada Precision Inc., Kusatsu, Japan). The experimental data were stored on a computer disc using an A/D and D/A transducer card (National Instruments Corporation, Austin, TX, USA).

## 3. Results and Discussions

### 3.1. Thermal Analysis and Electron Microscopy Analysis of Synthesized Powders

**Figure 4:**
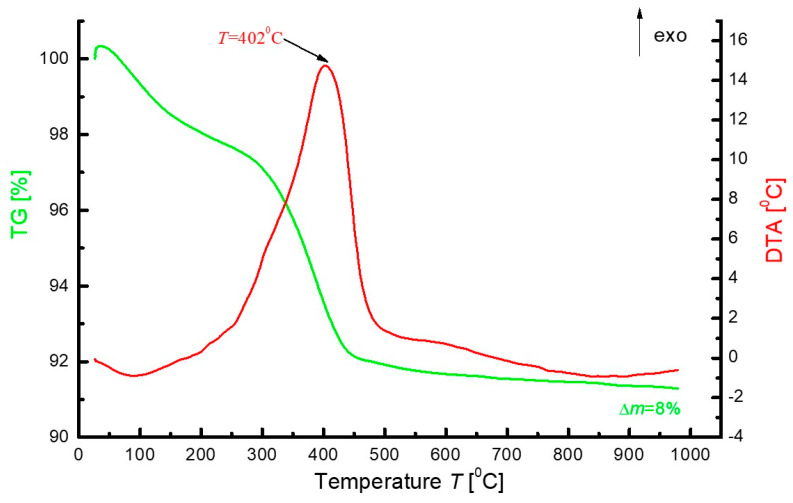
Thermal analysis data as exemplified by gel-combustion synthesized powders with the composition 9/65/35 PLZT:Pr^3+^0.3 (the same trend for all samples).

After fine grinding, the obtained gel-combustion powders of each PLZT:Pr^3+^ composition containing soot residues were subjected to thermogravimetric analysis (TGA) and differential thermal analysis (DTA). All samples showed the same trend, as shown in Figure 4. The TG curves show a two-stage mass loss: The first was about 2% and ranged from 30 to 250 °C, which may be due to gel decomposition and elimination of water content from the prepared sample, and the second was about 6% and occurred between 250 and 475 °C—accompanied by a strong exothermic effect—which can be interpreted as combustion of organic parts and soot formed in the first stage of synthesis. The presence of one exothermic peak in curve DTA also indicates the beginning of crystallization of the perovskite phase. Above the temperature of about 550 °C, the mass became fixed, which could indicate the stability of the composition. X-ray studies show that at temperatures of 600 °C, a perovskite phase of PLZT is formed, known to be stable until above 1000 °C when a lead is partially released.

The obtained thermogravimetric curves are similar to those obtained in the work [51], where they worked with citric acid as reaction fuel. Although the exothermic effect was interpreted as titanate–zirconate formation, this is incorrect. First, this exothermic effect is related to the rapid loss of mass in this temperature range (thus combustion or burning). Second, the enthalpy of the formation of zirconate titanates from oxides (generally composed of simple oxides) is very small and practically unmeasurable using this measurement technique.

### 3.2. SEM and TEM Analysis of 9/65/35 PLZT:Pr^3+^ Materials

**Figure 5:**
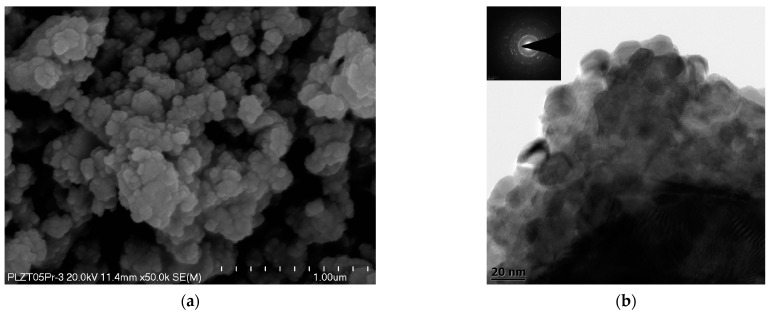
Sample SEM (**a**) and TEM (**b**) images of 9/65/35 PLZT:Pr^3+^0.5 powders obtained via gel-combustion synthesis (after calcination).

The SEM studies used to analyze the morphology of 9/65/35 PLZT:Pr^3+^ powders confirmed that regardless of chemical composition, the calcined powders created soft agglomerates, which revealed the submicron grain sizes ranging. For a more accurate characterization of the obtained powders, high-resolution HRTEM observations were used. Measurements showed that powder agglomerates consist of chemically homogeneous particles with regular isometric nanograins, with an average size of 10–20 nm, and a similar tendency was observed for all investigated powders. Sample SEM and TEM images obtained for the 9/65/35 PLZT:Pr^3+^0.5 powder are shown in Figure 5.

**Figure 6:**
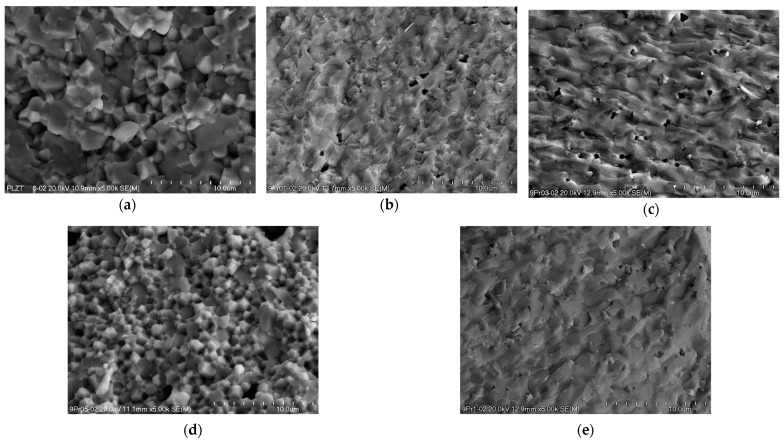
SEM micrographs (with mag. ×5.00 k) of sintered 9/65/35 PLZT:Pr^3+^ ceramics, with (**a**) 0; (**b**) 0.1; (**c**) 0.3; (**d**) 0.5; (**e**) 1 wt.% of dopants.

After sintering with the HUP method, high-quality compacts of ceramics were obtained. Each sample exhibited hardness and high density, which influenced the benefit of the studied material’s electrical and optical properties. Figure 6 shows SEM micrographs on the fracture surface (magnification 5000×), recorded for the undoped (Figure 6a) and praseodymium-doped (Figure 6b–e) 9/65/35 PLZT. The microstructure of pure PLZT and doped with 0.5 wt.% praseodymium ions exhibited well-shaped and well-crystallized, angular grains with distinct grain boundaries and devoid of porosity. Interestingly, fractures of the sample’s intercrystalline fracture mode were predominant in both cases. Figure 6a shows that the average grain size of 9/65/35 PLZT ceramic was 2–4 mm. In the 9/35/65 PLZT:Pr^3+^0.5 (Figure 6d), the microstructure showed smaller grains on average, ranging from submicron to 1–2 μm. The analysis of the other samples also revealed changes in the fracture surface of obtained ceramics with 0.1, 0.3, and 1 wt.% of praseodymium. All of them showed transcrystalline fracture (Figure 6b,c,e), and the microstructure was made up of fine, well-formed grains and a small amount of closed micropores in a compact solid manner. This behavior indicates high mechanical strength in the grain boundaries at the expense of the interior of the grains.

**Figure 7:**
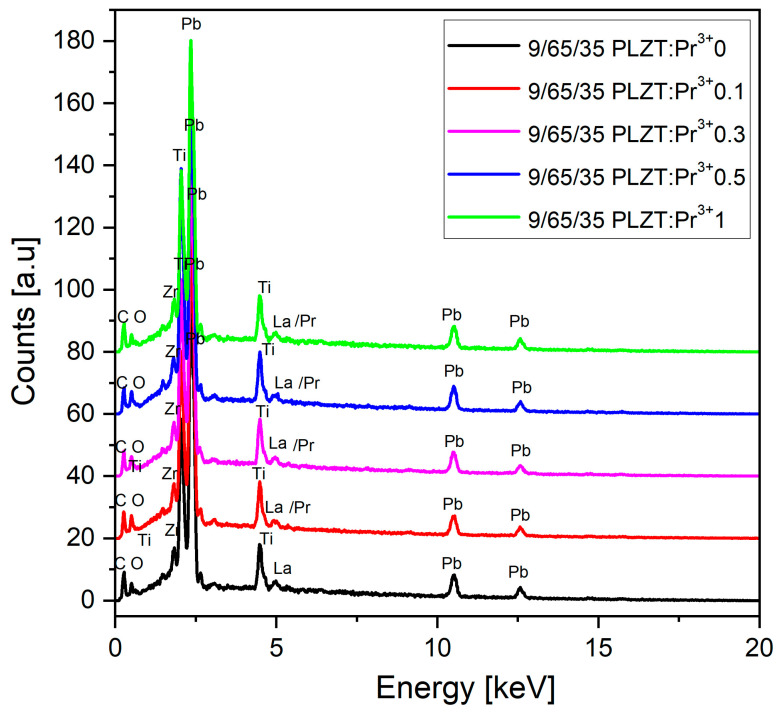
EDS spectra obtained for 9/65/35 PLZT:Pr^3+^ ceramic samples.

Results of the energy-dispersive spectroscopy (EDS) measurements confirmed the qualitative and quantitative chemical compositions of sintered 9/65/35 PLZT:Pr^3+^ ceramics. Each sample was characterized by high purity and homogeneity and the presence of only expected elements and manifested by intensities of the respective peaks (Figure 7).

The first part of Table 2 presents the values of the contents of the theoretically calculated elements in wt.% for each chemical composition of 9/65/35 PLZT:Pr^3+^. The second part of the table shows the average of the EDS analysis for the investigated sample on the five measurements in the randomly selected surface micro-areas (50 × 50 μm^2^). The differences between the obtained values and the theoretical stoichiometry are slight and are within the error limits of the method used. The EDS analysis showed no foreign elements, confirming the obtained materials’ high chemical quality.

### 3.3. X-ray Diffraction Analysis

**Figure 8:**
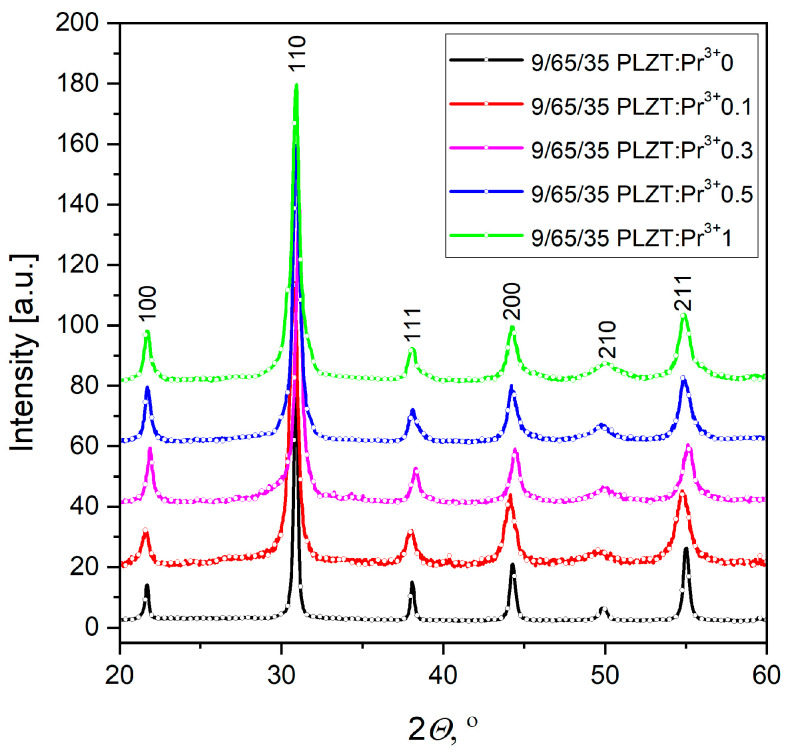
XRD pattern of 9/65/35 PLZT:Pr^3+^ ceramics, measured at room temperature.

The Rietveld refinement method, fixed into the X’Pert High Score (Panalytical, B.V) computer program, was used to calculate the elementary cell parameters. For the diffraction pattern fitting, a structure model from the ICDD database was used (PDF standards, N° 00-046-0336, 00-053-0785). The calculated values of the crystallographic parameters and the reliability factors determining the goodness of refinement (*R_p_*—reliability factor of the weighted patterns, *R_wp_*—reliability factor of the patterns, *R_exp_*—expected weighted profile factor) are summarized in Table 3. It is worth noting that the fitting parameters *R*_p_ and *R_wp_* are smaller than 9%, indicating that valid refinement results were obtained. Based on the analysis, it was found that each of the HUP-sintered (*T_s_* = 1100 °C) PLZT:Pr^3+^ bulk ceramics sample exhibits a complete perovskite phase and shows good homogeneity, as well as the formation of a single-phase compound with a pseudocubic structure with no pyrochlore (A_3_B_4_O_13_) (Figure 8). Also, the praseodymium doping does not change the symmetry of the perovskite lattice structure of 9/65/35 PLZT, regardless of the amount of dopant. It means that the Pr^3+^ ions have diffused into the host lattice, and the emerging lower symmetry may create conditions for ion off-centering and, thus, ferroelectricity of materials.

Table 3 also summarizes the lattice cell parameters of PLZT:Pr^3+^, which are used to determine the theoretical density of ceramic materials. This way, compared with the results obtained by the Archimedes method, it was found that the actual density of the materials obtained was 96–93% of the theoretical value.

### 3.4. 3D Fluorescence Maps, Excitation, and Emission Spectra

For all prepared samples, the luminescent spectra were measured to show how admixture of Pr^3+^ ions can influence the luminescent properties of 9/65/35 PLZT.

**Figure 9:**
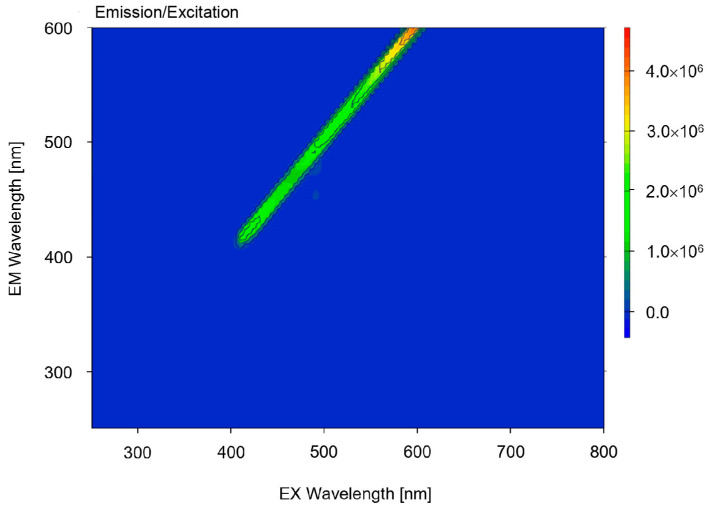
3D fluorescence spectra of 9/65/35 PLZT ceramics.

The 3D fluorescence spectra or excitation–emission matrices (EEMs) (Figure 9) were obtained from 250 to 600 nm in excitation at 5 nm intervals and from 250 to 800 nm in emission at 5 nm intervals. For undoped ceramics, no spectra were observed; only characteristics contour maps from the xenon lamp.

**Figure 10:**
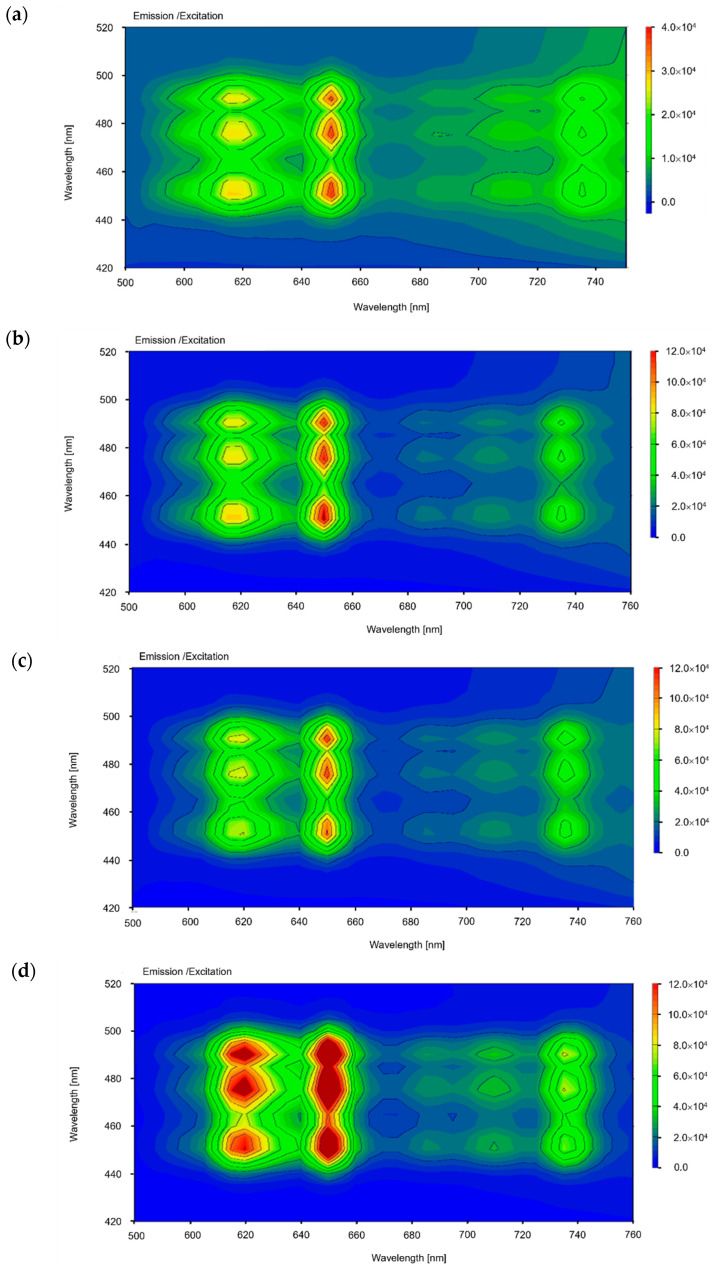
3D fluorescence spectra of 9/65/35 PLZT:Pr^3+^ ceramics with (**a**) 0.1, (**b**) 0.3, (**c**) 0.5, and (**d**) 1 wt.% of dopants.

In the case of 9/65/35 PLZT:Pr^3+^, 3D fluorescence spectra display fluorescence intensity in the contour “maps” formed in excitation–emission (Figure 10a–d), exhibiting fluorescence emission in the orange region of the visible spectrum with a peak at 650 nm.

**Figure 11:**
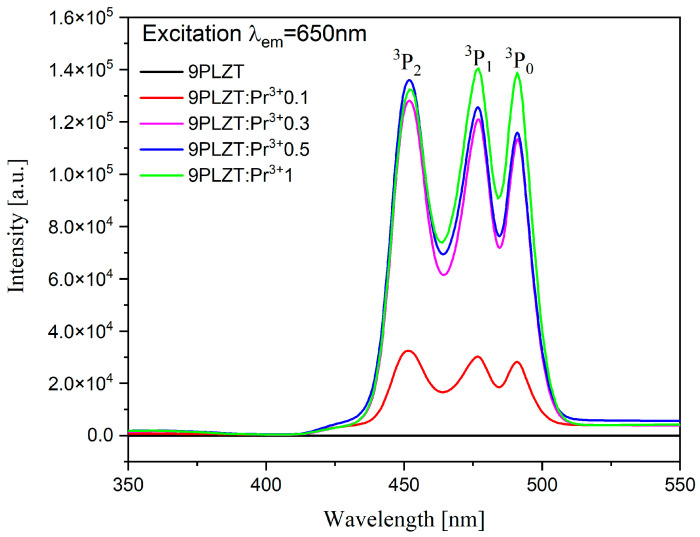
Excitation spectra for 9/65/35 PLZT:Pr^3+^ ceramics.

The excitation spectrum measured for the sintered samples is presented in Figure 11 and was recorded at λ_em_ = 650 nm. As mentioned above, the range was chosen based on the center of the most intense emission band corresponding to the ^3^P_0_→^3^F_2_ transition. One can see that the spectrum consists of three typical narrow bands associated with Pr^3+^ ions, which correspond to transitions originating from the ^3^H_4_ ground state to the ^3^P_2_. The 430 to 490 nm range is associated with the ^3^H_4_→^3^P_2_ (~450 nm), ^3^H_4_→^3^P_1_ (~476 nm), and ^3^H_4_→^3^P_0_ (~490 nm) transitions [52]. The bands are split because of the crystal-field effects. In addition, it can be seen that increasing the doping of Pr^3+^ ions from 0.1 to 1 wt.% in the 9/65/35 PLZT ceramic matrix also increased the excitation intensity.

**Figure 12:**
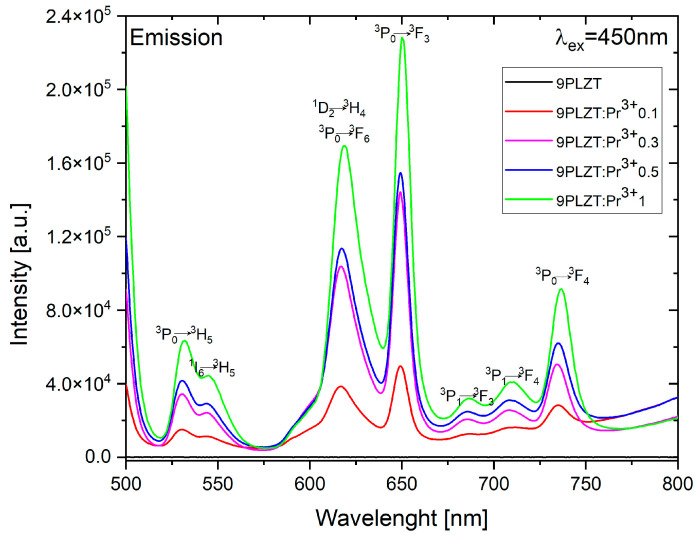
Emission spectra for 9/65/35 PLZT:Pr^3+^ ceramics.

Figure 12 shows emission spectra for Pr^3+^ ions in 9/65/35 PLZT ceramics. Such materials doped with praseodymium ions can excite red emission with UV and blue light radiation. In this measurement, investigated ceramic samples were excited at λ_exc_ = 450 nm, corresponding to the ^3^P_2_ level of Pr^3+^. This study obtained strong emission spectra consisting of green and red spectral ranges. The samples showed typical 4f transitions of Pr^3+^ ions, mainly from ^3^P_0_ and ^1^D_2_ excited states to ^3^F states. The low-intensity emission band in the green spectral region (530–550 nm) is associated with ^3^P_0_→^3^H_5_ and ^1^I_6_→^3^H_5_ transitions of Pr^3+^. The most intense emission lines are located in the red region.

Interestingly, in the presence of lanthanum ions in ceramics, emission bands shift towards the red, and doping with praseodymium ions resulted in a rich emission spectrum, especially in the bright and dark red range. Red emission lines are due to ^3^P_0_→^3^F_6_ and ^3^P_0_→^3^F_2_. The strongest emission effect, obtained at a wavelength of 650 nm, can be considered characteristic of the perovskite structure of PLZT:Pr^3+^ ceramics. For simple titanates, e.g., BaTiO_3_, SrTiO_3_, (Ba,Sr)TiO_3_, this effect occurs for lower wavelengths between 600 and 620 nm [53,54], and the obtained results once again confirm that the red emission dominates over blue emission similar to oxide host matrices. In general, the intensity of the emission lines depends on the activator concentration and technological conditions, as studied by the author in an earlier paper [40]. As already mentioned, the obtained 9/65/35 PLZT:Pr^3+^ samples showed a high level of ceramization due to the combination of the applied method of nanopowder synthesis and high-temperature densification of the samples using the HUP method. As a result, the nearest environment between optically active ions becomes more ordered, leading to an increase in the intensity of emission lines, even when using small amounts of dopant-activator ranging from 0.1 to 1 wt.% Pr^3+^, as demonstrated in this work.

**Figure 13:**
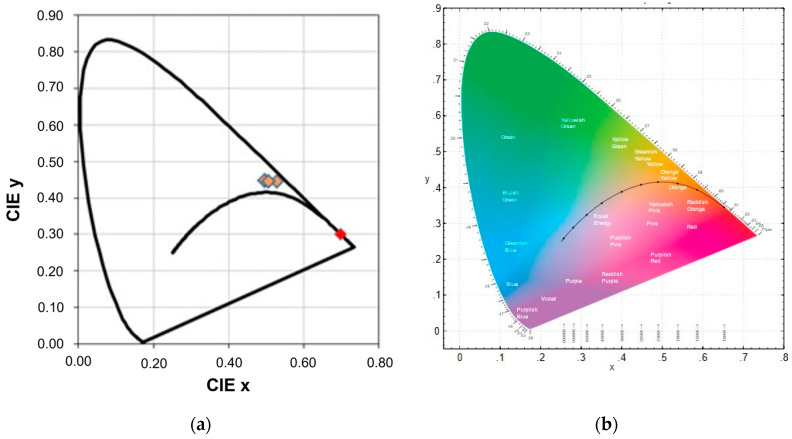
CIE chromaticity diagram (**a**) for investigated 9/65/35 PLZT:Pr^3+^ ceramics and (**b**) CIE standard color space [56].

We also studied the influence of the concentration of praseodymium dopant on the luminescence intensity for obtained samples. It stated that the content of praseodymium ions changes the shape of luminescence bands of optically active dopants. From this point of view, the Commission Internationale de I’Eclairage (CIE) chromaticity coordinates are calculated [55]. The diagram of CIE coordinates for 9/65/35 PLZT:Pr^3+^ ceramics is shown in Figure 13. The posted emission spectrum corresponds to the emitted color with CIE coordinates x = 0.496–0.529 and y = 0.445–0.446, i.e., the resulting PLZT:Pr^3+^ phosphor emits orange light, as well as reddish-orange color (x = 0.699, y = 0.301). There is, of course, the possibility of using filters and obtaining a red color.

### 3.5. Dielectric and Ferroelectric Properties

**Figure 14:**
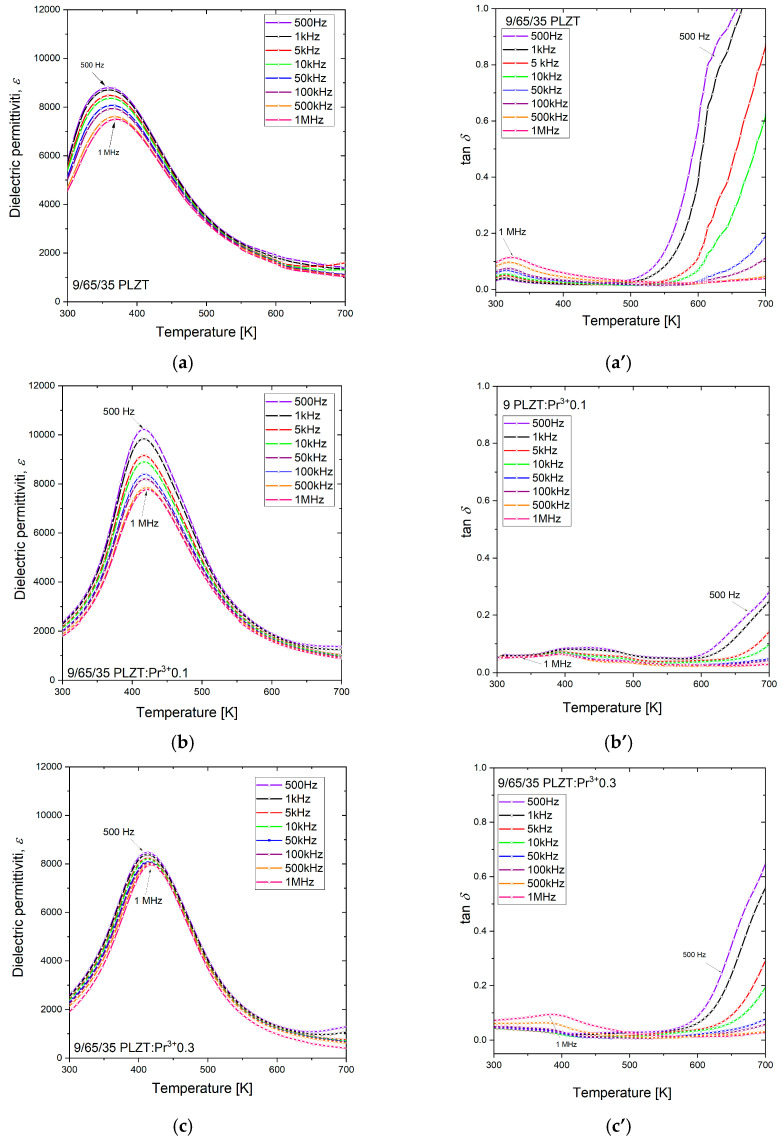

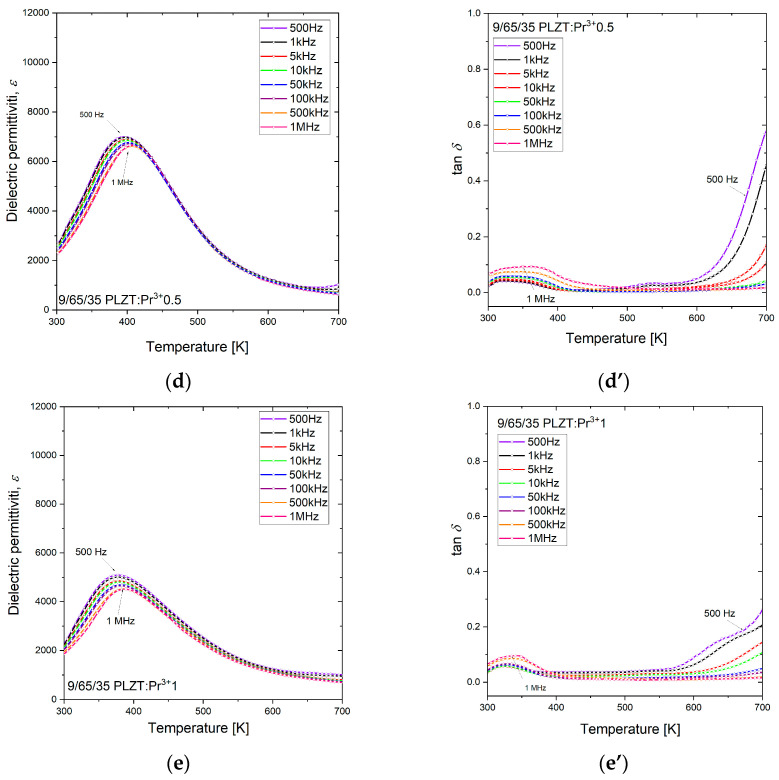
The dielectric constant (**a**–**e**) and the loss factor (**a’**–**e’**) versus temperature measured for a heating process at frequencies from 0.5 kHz to 1 MHz for 9/65/35 PLZT:Pr^3+^ ceramics.

Figure 14 shows the temperature-dependent variation of dielectric constant (*ε*) and dielectric loss tangent (tan*δ*) for praseodymium-doped 9/65/35 PLZT:Pr^3+^ ceramics over the temperature range of 300 K to 700 K, carried out at various frequencies of measurements field (*f* = 500 Hz–1 MHz).

The 9/65/35 PLZT composition is known for its relaxor ferroelectric behavior [14], as confirmed by the characteristic diffuse phase transition observed in the obtained material, which also manifests as a broad maximum in the change of dielectric constant with temperature (Figure 14a). A similar effect was observed in the ferroelectric–paraelectric transition that occurred over a wide temperature range for all other PLZT:Pr^3+^ samples (Figure 14b–e). In the same temperature range, frequency dispersion was also observed, manifested by a shift of these broad peaks of dielectric permittivities *ε*(*T*) toward higher temperatures as the frequency of the measurement field increases. This transition was characterized by the fact that the temperature maximum of *ε*(*T*) reaches higher values, while its *ε*_max_ value decreases with the increasing frequency of the measuring field.

As for dielectric losses, the opposite behavior was observed (Figure 14a’–e’). Due to space charge polarization, strong dielectric dispersion is observed below *T_m_*, while the tan*δ* value significantly increases with an increasing frequency above *T_m_*.

**Figure 15:**
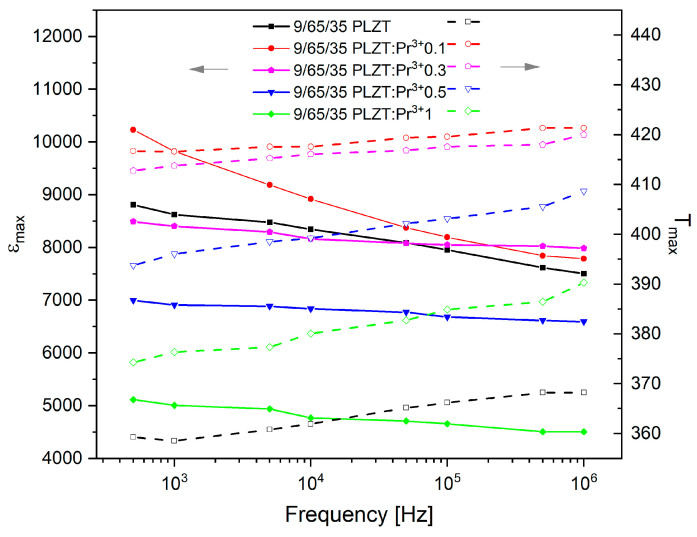
The curves of the maximum value of the electric permittivity Δ*ε*_max_ and the corresponding temperature Δ*T_max_* as functions of the frequency of measurement field.

To better illustrate the existence of a high-frequency dispersion, the curves of the maximum value dispersion of the electric permittivity Δ*ε*_m_ and the corresponding temperature Δ*T_m_* in the function of frequency are presented in Figure 15. Subsequently, to compare the sizes of both dispersions, the value of degrees of dispersion was calculated as follows:

∆*ε*_m_ = *ε*_m500Hz_ − *ε*_m1MHz_ (5)

∆*T*_m_ = *T*_m1MHz_ − *T*_m500Hz_ (6)

Data analysis shows that a small admixture of praseodymium ions (0.01 wt.%) causes a significant increase in Δ*ε*_max_ and the reduction in Δ*T_m_*. With the next increase in the content of praseodymium ions to 1 wt.%, an increase in both parameters confirms the increasing frequency dispersion. However, the values of Δ*T_m_* differ significantly from those obtained for classic relaxors, such as 8/65/35 PLZT, for which Δ*T*_m_ = 25 K [57]. The determined values are summarized in Table 3.

**Figure 16:**
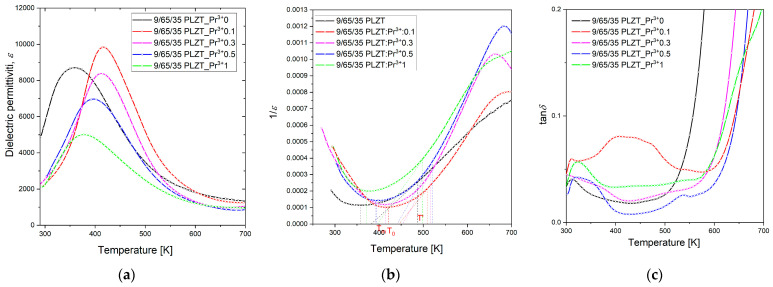
Temperature dependence of the (**a**) dielectric permittivity, (**b**) reciprocal of the electric permittivity, and (**c**) loss tangent *δ*, measured at *f* = 1 kHz for the 9/65/35 PLZT:Pr^3+^ ceramics.

The low-frequency (1 kHz) temperature dependence on the dielectric constant measured for 9/65/35 PLZT:Pr^3+^ ceramics demonstrates how the used praseodymium dopants influenced the dielectric behaviors of the PLZT ceramics matrix (Figure 16, Table 4).

The addition of praseodymium ions as an admixture (0.1 wt.%) initially caused a shift toward higher temperatures, reaching the maximum dielectric permittivity (*T_m_*). Further increase in doping (0.3–1 wt.%) results, in turn, in a gradual decrease in the phase transition temperature and significantly decreases the maximum permittivity value (*ε*_m_). In [58], the defect model has been proposed to explain the relationship between grain size and the properties of ceramic materials. It was described that when the grain boundaries increase as the grain size decreases, the lattice defects increase, contributing to a decrease in the dielectric constant. Because the ferroelectric domain’s structure is formed close to the Curie temperature, lattice distortion energy is released by the domain’s formation.

Additionally, it was observed that praseodymium influences the broadening of the maximum dielectric permittivity, which is typical for *ε*(T) behavior of ferroelectric relaxor materials (Figure 16a). The shape of these characteristics indicates the ability to apply the modified Curie–Weiss law in a wide range of temperatures above the *T_m_* temperature (temp. Curie). For this reason, the temperature *T*’, at which the Curie–Weiss law is applied, was determined based on the 1/*ε*(T) characteristic (Figure 16b). *T_m_*, *T*_0_, and *T*’ values strongly depend on praseodymium concentration, and the parameters k_1_, k_2_, and k_3_ (Table 3) calculated from them highlight how the degree of blurring of the occurring phase transition changes. It indicates a change in the distribution of the ferroelectric microregion within the material, wherein an increasing part transforms from the ferroelectric to the paraelectric phase.

It was observed that 9/65/35 PLZT:Pr^3+^ samples have relatively low dielectric loss factor values up to about ~580 °C (Figure 16c). At room temperature, the tan*δ* values are below 0.08 and increase rapidly above 510 K, related to the increased electrical conductivity of samples at higher temperatures. At the same time, the phase transition from ferroelectric to paraelectric phase takes place at higher temperatures (although the *T_m_* temperature gets smaller), compared to undoped 9/65/35 PLZT.

Subsequently, after identifying a diffuse character of the phase transition of 9/65/35 PLZT:Pr^3+^ ferroelectric ceramics, the modified Curie–Weiss law was also used to evaluate the degree of its diffusivity.

(7) $$\frac{1}{\varepsilon }-\frac{1}{\varepsilon_{m}}=C{\left(T-T_{m}\right)}^{\gamma }$$

where *ε* is the dielectric permittivity constant, *ε_m_* is the maximum value of a dielectric permittivity constant, *T_m_* is the temperature of the value dielectric permittivity maximum, C represents Curie constant, and *γ* represents the parameters indicating the degree of blur of the phase transition and ferroelectric relaxation behavior.

**Figure 17:**
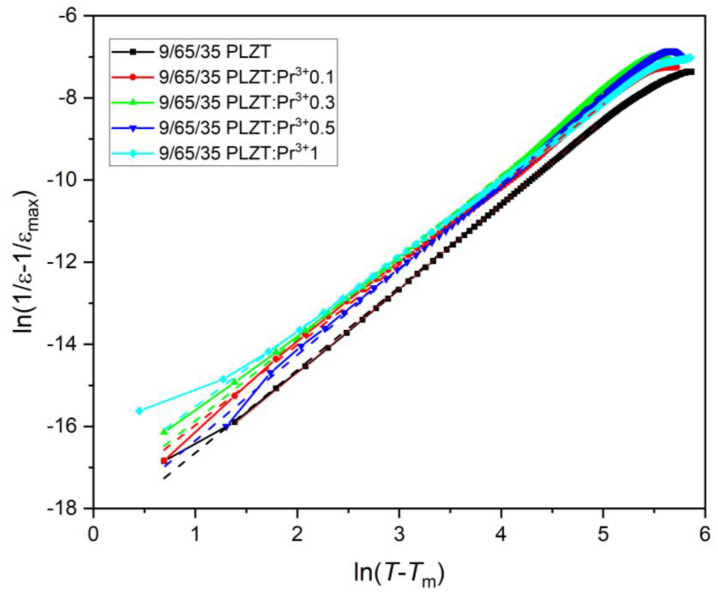
Plots of ln(1/*ε* − 1/*ε_m_*) vs. ln(*T* − *T_m_*) at temperatures higher than *T_m_* for the 9/65/35 PLZT:Pr^3+^ ceramics (*f* = 1 kHz).

In this case, *γ* = 1 indicates normal Curie–Weiss behavior, while *γ* = 2 represents a relaxor phase transition. The *γ* parameter can be calculated from the slope of the graph plotted between ln(1/*ε* − 1/*ε*_max_) and ln (*T* − *T_m_*) [37,59] (Figure 17), and all *γ* values less than 1 kHz are given in Table 3.

In the case of the composition with 0.1 wt.% Pr^3+^, the calculated *γ* decreases compared to the *γ* of undoped 9/65/35 PLZT ceramics. It reveals that the ferroelectric transition becomes more ordered and slightly reduces the relaxor property. However, the obtained data increased when the dopant content increased from 0.3 to 1 wt.%. It confirms the influence of the admixture of praseodymium on increasing the degree of transformation and thus strengthening the relaxor phase transition of the 9/65/35 PLZT:Pr^3+^ materials. For the 1 wt.% of dopant, *γ* achieved similar values to its original value for pure PLZT ceramic matrix.

The structural studies in Section 3.3 showed that the PLZT:Pr^3+^ materials we obtained have a pseudocubic structure. It is well known that materials that exhibit the cubic phase are generally non-ferroelectric, while ferroelectric materials typically have a non-cubic crystal structure that gives rise to these properties. From a physical point of view, it is difficult to explain that ferroelectricity can occur in cubic symmetry. However, this occurs in lead-based and lead-free materials. In a study of BiFeO_3_-BaTiO_3_ ceramic systems, Wang et al. [60] suggested that local structures can contribute to the ferroelectric response. The model proposed by the author implies that those local polar regions distort in the direction of the applied field within multiple local symmetries (pseudosymmetry) without long-range correlation. The nanodomain formation in the BF−BT system is associated with Bi^3+^ ions off-centering [61], because bismuth has single-pair electrons that can lead to asymmetric distortions and off-center shifts. Therefore, ferroelectric behavior in lead-free materials is associated with realizing structure nanodomains by the off-center Bi ions [62].

The PLZT ceramic materials have a cubic crystal structure at room temperature, and the presence of Pb^2+^ ions can cause a non-centrosymmetric displacement of the Pb ion from its ideal position within the perovskite unit cell. This off-centering causes a net polarization and can lead to ferroelectric properties due to subtle distortions from perfect cubic symmetry. Notably, the presence of a pseudocubic structure or off-centering of ions on its own does not guarantee ferroelectricity. The structure and chemistry are crucial in determining the material’s properties [63].

When dealing with relaxor materials, it is important to consider that their behavior contrasts with traditional ferroelectric materials, where polarization is consistently aligned throughout the crystal structure. Relaxors, such as PLZT ceramics, are distinguished by the absence of long-range order in polarization. These materials comprise nanoscale domains known as polar nanoregions (PNRs). Within these PNRs, local polarizations are dynamic and contribute to the broadened phase transition and the material’s unique dielectric response, which depends on frequency. The polarization in each nanoregion is locally coherent but varies across different regions. Despite being only nanometers in scale, these regions are pivotal to the behavior of relaxor ferroelectrics, primarily due to the transient and variable alignment of local dipoles within these nanoregions [64]. It is commonly believed that the relaxor phenomenon originates from compositional fluctuations in microregions of about 10 nm. Defects in the crystal lattice can disrupt the local electric environment, affecting the alignment of dipoles and thus influencing the ferroelectric characterization.

**Figure 18:**
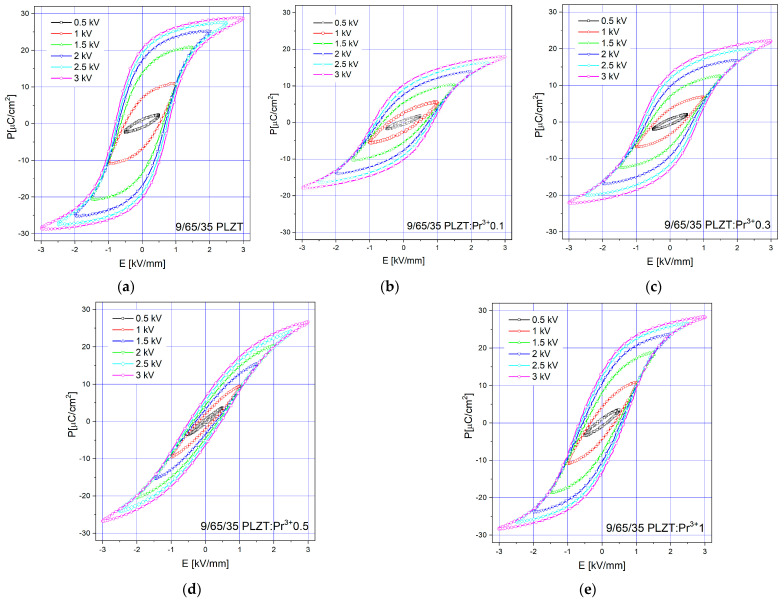
Polarization versus electric field (*P*(*E*)) hysteresis loops as a function of applied electric field investigated for 9/65/35 PLZT:Pr^3+^ ceramics, measured at room temperature.

Subsequently, in the next step of investigations, the ferroelectric behavior of all samples was measured, and the basic parameters were determined. Figure 18 shows the room temperature P-E hysteresis loops of the 9/65/35 PLZT:Pr^3+^ ceramics, examined at a frequency of *n* = 1 Hz, under various applied electric fields (0–3 kV/mm^2^). Several factors influence the shape and parameters of the hysteresis loop, i.e., composition, microstructure, crystallographic phase, domain structure and size, temperature, defects, vacancies, or impurities. Understanding and optimizing these influencing factors are key aspects in the development of advanced ferroelectric materials and devices.

All investigated materials exhibit a typical slim-like hysteresis loop for relaxors. It was observed that with the increase in the value of the electric field, the remnant polarization (*P_R_*)_,_ saturation polarization (*P_S_*), and coercive field (*E_C_*) increase consequently. Regardless of chemical composition, the hysteresis loops are well-saturated and fully developed, partly confirming that the obtained materials have excellent properties.

Comparing the shape of the hysteresis loop obtained for pure 9/65/35 PLZT, the introduction of 0.1 wt.% dopant causes a slight change, like the loop, to be more quadratic by reducing the *Ps* and *P_R_* parameters. However, a further increase in praseodymium doping from 0.3 to 1 wt.% results in additional changes, leading to an increase in *P_S_* and *P_R_*, although at a relatively low electric field *E_C_*. At the same time, the slimmest loop among the tested samples is characterized by ceramics with 0.5 wt.% doping of Pr^3+^ ions.

**Figure 19:**
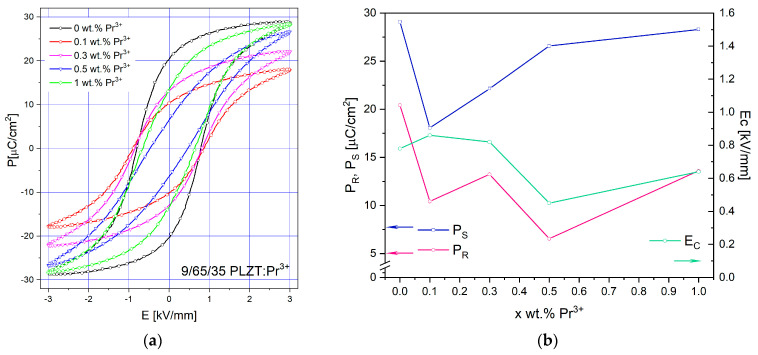
Ferroelectric P-E loops of 9/65/35 PLZT:Pr^3+^ ceramics (**a**,**b**) and ferroelectric parameters (*Ps*, *P_R_*, and *Ec*) as a function of praseodymium amount in the 9/65/35 PLZT:Pr^3+^ ceramics.

To better illustrate the influence of praseodymium dopant on the ferroelectric properties of 9/65/35 PLZT, Figure 19a summarizes the P(E) electric hysteresis loops at room temperature and frequency of 1 Hz and a maximum of electric field E = 3 kV/mm. Figure 19b shows the evaluation of ferroelectric parameters, including saturated polarization, remnant polarization, and coercive field, as a function of the amount of Pr^3+^ ions in the ceramic matrix. Such a typical character indicates that the PLZT:Pr^3+^ ceramics are rather soft ferroelectric materials. Doping can also result in polarization and domain switching, which causes induced strain in ferroelectric materials, and it is feasible to tailor the poling condition to obtain interesting piezoelectric properties [28]. The ferroelectric parameters *P_R_*, *E_C_*, and *P_S_* are given in Table 5.

The spontaneous polarization (*P_s_*) is a critical parameter in determining the suitability and efficiency of ferroelectric materials for various applications, including memory devices, sensors, actuators, and other electronic components. The configuration and size of domains can impact the *P_S_*. It is worth noting that larger, well-aligned domains typically contribute to higher spontaneous polarization value. Defects in the crystal lattice can disrupt the local electric environment, affecting the alignment of dipoles and thus influencing the spontaneous polarization. Depending on their nature and interaction with the crystal lattice, certain dopants or defects can either enhance or reduce the spontaneous polarization [65].

The remanent polarization is another important characteristic in applying ferroelectric materials in non-volatile memory devices, capacitors, and other electronic components, where retaining polarization after removing an external field is crucial. Just like with spontaneous polarization, the material’s chemical composition and crystal structure play an important role in determining its value. Smaller grain sizes often lead to lower remanent polarization due to the increased grain boundary area, which can impede domain wall motion. Defects in the crystal lattice and impurities can pin domain walls, making it more difficult for the material to switch polarization. This pinning can increase the remanent polarization by preventing the domains from returning to their unpolarized state after removing the external field. Larger, well-aligned domains usually contribute to a higher remanent polarization. The ease with which domains can be reoriented in an electric field also influences the *P_R_* [65].

The coercive field in a dielectric hysteresis loop is an important parameter representing the intensity of the electric field required to switch the material’s polarization from one direction to another. In ferroelectric materials, domains are regions where the polarization is uniformly aligned. In polycrystalline ferroelectrics, the grain size can influence the coercive field. Smaller grains often lead to lower coercive fields because of the increased grain boundary area, which can act as nucleation sites for domain switching. The size and distribution of these domains can significantly affect the coercive field. Finer domain structures can reduce the coercive field because smaller domains can be easier to switch [65].

The room temperature hysteresis loop of all examined samples is shown in Figure 19a. One can see that the loop area of the PLZT:Pr^3+^ is smaller than that of 9/65/35 PLZT. The loop gets slender for the higher doping concentration of praseodymium (0.5 wt.%). The value of coercive field (*E_c_*) and remanent polarization (*P_R_*) of the PLZT:Pr^3+^ compounds first decreases with increasing dopant amount and then increases for higher doping (1.0 wt.%) (Table 4). Several factors could be at play to explain this phenomenon. Firstly, the whole behavior could be linked to subtle variations in the composition of 9/65/35 PLZT as the amount of Pr^3+^ in the host lattice changes. Even minor changes in the composition can significantly affect the material’s ferroelectric properties. The material’s microstructure, including defects, can influence its dielectric and ferroelectric properties. As Pr increases, the microstructure may undergo changes that impact ferroelectric behavior. Changes in domain structure, including domain size, domain wall mobility, and domain pinning, can affect the shape of P-E hysteresis loops and the ferroelectric response. Complex interactions between ions (Pb, La, Zr, Ti) and the role of Pr doping or variations in their local environments could contribute to the observed behavior.

## 4. Conclusions

The work here discusses a detailed study of the effect of doping with praseodymium ions on the physical properties of ferroelectric 9/65/35 PLZT ceramics. The low-temperature gel-combustion method synthesized lead-lanthanum zirconate titanate, modified with various amounts of Pr^3+^ dopant (0–1 wt.%).

The prepared powders were investigated for the thermal stability and the microscopic analysis to control morphology. TGA/DTA confirms that the crystal formation appears to take place above 475 °C. SEM/TEM observations confirmed the high purity and quality of obtained nanopowders, which tended to form soft agglomerates. All samples were then consolidated by the hot uniaxial pressing method. As a result, homogeneous ceramic materials in packing, grain sizes, and phase composition were obtained. This affected the high quality of the obtained ceramic sinters because all samples were characterized by high hardness, negligible closed porosity, and thus, high density. SEM tests confirmed that the samples were characterized by a compact, fine-grained microstructure, regardless of the chemical composition. EDS results showed high quantitative and qualitative composition and material homogeneity, free of contamination and other foreign elements.

As XRD analysis has shown, both undoped and praseodymium-doped 9/65/35 PLZT materials show a pseudocubic perovskite single-phase. In a pseudocubic structure, the subtle distortions from a perfect cubic symmetry can allow the ions to occupy off-center positions, giving rise to a permanent electric dipole moment, a prerequisite for ferroelectricity. Also, the praseodymium doping in 0–1 wt.% does not change the symmetry of the perovskite lattice structure of 9/65/35 PLZT ceramics. It means that the Pr^3+^ ions have diffused into the host lattice. This behavior promotes a transformation of the ceramic ferroelectric into a ceramic phosphor without losing its existing properties.

The luminescence activity of the obtained PLZT:Pr^3+^ samples has been visualized using 3D fluorescence maps. Excitation and emission spectra of praseodymium ions in 9/65/35 PLZT ceramics were measured, and it was observed that intensities changed with activator concentration. Several emission bands due to ^3^P_0_-^3^H_4_, ^1^D_2_-^3^H_4_, ^3^P_0_-^3^H_4_, and ^3^P_0_-^3^F_2_ transitions of praseodymium ions are observed under excitation at λ_exc_ = 450 nm. It was found that the most intense emission lines are located in the red region, which was also calculated by I’Eclairage (CIE) chromaticity coordinates.

Praseodymium doping also affects the dielectric and ferroelectric characteristics of 9/65/35 PLZT ceramics. The addition of a small amount of 0.1 wt.% of admixture initially causes a shift toward higher temperatures (*T_m_*) of the maximum dielectric permittivity compared to undoped composition. Further increase in doping (0.3–1 wt.%) results, in turn, causes a gradual decrease in the phase transition temperature and significantly decreases the maximum permittivity value (*ε*_m_). Additionally, praseodymium influences the broadening of the maximum dielectric permittivity, which is typical for the ferroelectric relaxor materials’ behavior. The tan*δ* values at room temperature are below 0.1 and increase rapidly above the Curie temperature *T*_m_, related to the increased electrical conductivity of samples at higher temperatures, regardless of composition.

The appearance of a hysteresis loop gives a reliable confirmation of the ferroelectric properties of the material. The 9/65/35 PLZT:Pr^3+^ materials exhibit a typical relaxor slim-like hysteresis loop. It was observed that with the increase in the value of the electricity, the remnant polarization (*P_R_*), saturation polarization (*P_S_*), and coercive field (*E_C_*) increase consequently for each composition. Regardless of chemical composition, the hysteresis loops are well-saturated and fully developed, partly confirming that the obtained materials have excellent relaxor-ferroelectric properties. Interestingly, introducing 0.1 wt.% dopant causes a slight change, like the loop being more quadratic by reducing the Ps and P_R_ parameters. However, a further increase in dopant from 0.3 to 1 wt.% results in additional changes in the values of P_S_ and P_R_, although at a relatively low electric field EC. Among studied materials, the slimmest loop has been exhibited by ceramics with 0.5 wt.% doping of Pr^3+^ ions. This ceramic composition shows well-shaped, small grain sizes of ∼1.2 μm, with clear and visible grain boundaries, uniformity in grain sizes, and low porosity, thereby showing optimal electrical properties. Smaller grains often lead to lower coercive fields because of the increased grain boundary area, which can act as nucleation sites for domain switching.

## Figures and Tables

**Table 1:** Materials designation as a function of the praseodymium amount.

Chemical Formula	Designed
(Pb_0.91_La_0.09_)(Zr_0.65_Ti_0.35_)_0.9775_O_3_	9/65/35 PLZT:Pr^3+^0
(Pb_0.91_La_0.09_)(Zr_0.65_Ti_0.35_)_0.9775_O_3_ + Pr^3+^0.1 wt.%	9/65/35 PLZT:Pr^3+^0.1
(Pb_0.91_La_0.09_)(Zr_0.65_Ti_0.35_)_0.9775_O_3_ + Pr^3+^0.3 wt.%	9/65/35 PLZT:Pr^3+^0.3
(Pb_0.91_La_0.09_)(Zr_0.65_Ti_0.35_)_0.9775_O_3_ + Pr^3+^0.5 wt.%	9/65/35 PLZT:Pr^3+^0.5
(Pb_0.91_La_0.09_)(Zr_0.65_Ti_0.35_)_0.9775_O_3_ + Pr^3+^1 wt.%	9/65/35 PLZT:Pr^3+^1

**Table 2:** Theoretical and determined chemical composition of 9/65/35 PLZT:Pr^3+^ ceramics.

**Compositions/Elements**	**The Theoretical Content of Elements (wt.%)**					
	**Pb**	**La**	**Zr**	**Ti**	**O**	**Pr**
**9PLZT**	58.305	3.866	17.923	5.064	14.842	---
**9PLZT:Pr^3+^0.1**	58.247	3.862	17.905	5.059	14.827	0.099
**9PLZT:Pr^3+^0.3**	58.131	3.854	17.869	5.049	14.797	0.299
**9PLZT:Pr^3+^0.5**	58.015	3.847	17.834	5.039	14.768	0.497
**9PLZT:Pr^3+^1**	57.728	3.821	17.746	5.014	14.695	0.996
**Compositions/Elements**	**Measured Content of Elements (wt.%)**					
	**Pb**	**La**	**Zr**	**Ti**	**O**	**Pr**
**9PLZT**	59.006	3.780	17.292	5.087	14.835	---
**9PLZT:Pr^3+^0.1**	58.462	3.659	17.979	5.065	14.749	0.086
**9PLZT:Pr^3+^0.3**	57.977	3.943	17.324	5.561	15.195	0.230
**9PLZT:Pr^3+^0.5**	58.128	3.772	17.689	5.080	14.790	0.541
**9PLZT:Pr^3+^1**	57.899	3.580	17.510	5.102	14.995	0.914

**Table 3:** The lattice parameters of Rietveld fitting, obtained for 9/65/35 PLZT:Pr^3+^ ceramics: diffraction pattern fitting factors: R*_p_*—primary, *R_wp_*—weighed, *R_exp_*—experimental; *a*_0_, *b*_0_, *c*_0_—parameters of unit cell; *V*—unit cell volume; *ρ_theor_*—theoretical density.

Parameters/Samples	9/65/35 PLZT	9PLZT:Pr^3+^0.1	9PLZT:Pr^3+^0.3	9PLZT:Pr^3+^0.5	9PLZT:Pr^3+^1
*a*_0_ (nm)	4.074	4.041	4.052	4.063	4.079
*b*_0_ (nm)	4.074	4.041	4.052	4.063	4.079
*c*_0_ (nm)	4.074	4.041	4.052	4.063	4.079
*V* × 10^−30^ (nm)	67.62	65.99	66.53	67.07	67.53
*R_p_* (%)	6.02	6.43	6.09	6.99	6.09
*R_wp_* (%)	8.14	8.92	8.11	8.56	8.11
*R_exp_* (%)	4.39	4.13	4.72	4.34	4.53
ρ × 10^−3^ (g/cm^3^)	7.66	7.21	7.18	7.52	7.38
ρ/ρ_theor_ (%)	96	94	93	95	93

**Table 4:** Parameters of the 9/65/35 PLZT:Pr^3+^ ceramics (where RT—room temperature).

Parameters/Samples	9/65/35 PLZT	9PLZT:Pr^3+^0.1	9PLZT:Pr^3+^0.3	9PLZT:Pr^3+^0.5	9PLZT:Pr^3+^1
Δ*ε_m_*	1305.86	2445.0	504.93	403.95	609.5
Δ*T_m_*	8.92	4.66	7.22	14.91	16.01
*εRT*	4889.64	2156.19	2332.80	2139.15	2138.10
*ε_m_*	8692.69	9860.78	8400.07	6974.50	5025.47
*T_m_* (K)	358.51	418.48	414.01	394.93	374.76
*T*_0_ (K)	380.16	446.89	454.70	442.48	396.81
*T*’ (K)	487.09	509.09	516.12	510.55	497.63
k_1_ = *εm*/*εRT*	1.78	4.57	3.60	3.26	2.35
k_2_ = *T*’ − *T*_0_ (K)	106.93	63.09	58.42	68.07	100.02
k_3_ = *T*’ − *Tm* (K)	128.58	90.61	102.11	115.62	122.87
*γ*	2.08	1.84	1.95	2.00	2.02

**Table 5:** Ferroelectric parameters of 9/65/35 PLZT:Pr^3+^ ceramics.

Parameters/Samples	9/65/35 PLZT	9PLZT:Pr^3+^0.1	9PLZT:Pr^3+^0.3	9PLZT:Pr^3+^0.5	9PLZT:Pr^3+^1
*P_s_* (mC/cm^2^)	29.09	18.07	22.15	26.59	28.30
*P_R_* (mC/cm^2^)	20.44	10.42	13.24	6.55	13.45
*E_C_* (kV/mm)	0.78	0.86	0.82	0.45	0.64

## Data Availability

Data are contained within the article.

## References

[1] Uchino K. (2015). Glory of piezoelectric perovskites. Sci. Technol. Adv. Mater..

[2] Haertling G.H. (2002). Piezoelectric and electrooptic ceramics. Ceramic Materials for Electronics, Processing, Properties and Applications.

[3] Haertling G.H. (1999). Ferroelectric ceramics: History and technology. J. Am. Ceram. Soc..

[4] Płońska M., Czekaj D., Surowiak Z. (2003). Application of the sol-gel method to the synthesis of ferroelectric nanopowders (Pb_1−x_La_x_)(Zr_0.65_Ti_0.35_)_1−0.25x_O_3_, 0.06 ≤ x ≤ 0.1. Mater. Sci..

[5] Murakami S., Morita M., Herren M., Sakurai T., Rau D. (2000). Near-infrared luminescence and spectral anomaly in PLZT ceramics doped with Nd^3+^, Er^3+^, Yb^3+^ and Cr^5+^ ions at low temperatures. J. Lumin..

[6] Boccaccini R.A., Silva D.D. (2008). Industrial Developments in the Field of Optically Transparent Inorganic Materials: A Survey of Recent Patents. Recent Pat. Mater. Sci..

[7] Qiao L., Ye Q., Gan J.L., Cai H.W., Qu R.H. (2011). Optical characteristics of transparent PMNT ceramic and its application at high speed electro-optic switch. Opt. Commun..

[8] Samanta S., Muralidhar M., Sankaranarayanan V., Sethupathi K., Ramachandra Rao M.S., Murakami M. (2017). Band gap reduction and redshift of lattice vibrational spectra in Nb and Fe co-doped PLZT. J. Mater. Sci..

[9] Wei Z., Huang Y., Tsuboi T., Nakai Y., Zeng J., Li G. (2012). Optical characteristics of Er^3+-^doped PMN–PT transparent ceramics. Ceram. Int..

[10] Kyômen T., Sakamoto R., Sakamoto N., Kunugi S., Itoh M. (2005). Photoluminescence Properties of Pr-Doped (Ca,Sr,Ba)TiO_3_. Chem. Mater..

[11] Durruthy-Rodríguez M.D., Yáñez-Limón J.M. (2011). Photoluminescence in Doped PZT Ferroelectric Ceramic System.

[12] Ramovatar, Coondoo I., Satapathy S., Kumar N., Panwar N. (2018). Dielectric, Piezoelectric Enhancement and Photoluminescent Behavior in Low Temperature Sintered Pr-Modified Ba_0.85_Ca_0.15_Zr_0.1_Ti_0.9_O_3_ Ceramics. J. Electron. Mater..

[13] Praveenkumar B., Kumar H.H., Kharat D.K., Murty B.S. (2008). Investigation and characterization of La-doped PZT nanocrystalline ceramic prepared by mechanical activation route. Mater. Chem. Phys..

[14] Botero E.R., Eiras J.A., Guo R., Bhalla A., Garcia D. (2011). Members of Lanthanide Family as Dopants in Relaxor PLZT Ceramics. Itegr. Ferroelectr..

[15] Okazaki K., Masuda M., Tashiro S., Ishibashi S. (1978). Defect structure and properties of electro-optic PLZT ceramics. Ferroelectrics.

[16] Singh R., Goel T.C., Chandra S. (2008). RF magnetron sputtered La^3+^-modified PZT thin films: Perovskite phase stabilization and properties. Mater. Chem. Phys..

[17] Shannigrahi S.R., Choudhary R.N.P., Acharya H.N. (1999). Effect of Er doping on structural and dielectric properties of sol-gel prepared PZT ceramics. Mater. Res. Bull..

[18] De Camargo A.S.S., Jacinto C., Nunes L.A.O., Catunda T., Garcia D., Botero É.R., Eiras J.A. (2007). Effect of Nd^3+^ concentration quenching in highly doped lead lanthanum zirconate titanate transparent ferroelectric ceramics. J. Appl. Phys..

[19] Zheng Z., Li X., Liu J., Feng Z., Li B., Yang J., Li K., Jiang H., Chen X., Xie J. (2008). Optical properties of Er^3+^/Yb^3+^-codoped transparent PLZT ceramic. Physica B.

[20] Ballato J., Esmacher R., Schwartz R., Dejneka M. (2000). Phonon sideband spectroscopy and 1550 nm luminescence from Eu^3+^ and Er^3+^-doped ferroelectric PLZT for active electro-optic applications. J. Lumin..

[21] De Camargo A.S.S., de ONunes L.A., Santos I.A., Garcia D., Eiras J.A. (2004). Structural and spectroscopic properties of rare-earth *(*Nd3+*,* Er3+*,*and Yb3+*)*doped transparent lead lanthanum zirconate titanate ceramics. J. Appl. Phys..

[22] Osińska K., Płońska M., Marzec A. (2016). Application of the sol-gel method at the fabrication of PLZT:Yb^3+^ ceramics. Arch. Metall. Mater..

[23] Garcia D., Menegazzo B.A., Eiras J.A. (1992). Stoichiometric variation and dielectric properties in 9/65/35 PLZT ceramics. Ceramica.

[24] Tunaboylu B., Harvey P., Esener S.C. (1998). Characterization of dielectric and electro-optic properties of PLZT 9/65/35 films on sapphire for electro-optic applications. IEEE Trans. Ultrason. Ferroelectr. Freq. Control.

[25] Chen F., Smith P.W., Dobson P.J. Domain switching of PLZT 9/65/35 in ferroelectric electron emission. Proceedings of the 2000 Eighth International Conference on Dielectric Materials, Measurements and Applications (IEE Conf. Publ. No. 473).

[26] Kutnjak Z., Bobnar V., Filipič C., Levstik A. (2001). Glassy properties of 9/65/35 PLZT ceramics. Ferroelectrics.

[27] Khodorov A., Gomes M.J.M. (2006). Optical Properties of PLZT 9/65/35 Thin Films on ITO-Coated Glass Substrate. Mater. Sci. Forum.

[28] Somwan S., Ngamjarurojana A., Limpichaipanit A. (2016). Dielectric, ferroelectric and induced strain behavior of PLZT 9/65/35 ceramics modified by Bi_2_O_3_ and CuO co-doping. Ceram. Int..

[29] De Camargo A.S.S., Botero E.R., Nunes L.A.O., Lente M.H., Santos I.A., Andreeta E.R.M., Garcia D., Eiras J.A. (2004). Ferroelectric ceramic materials as hosts of laser active ions: Structural, microstructural and spectroscopic characteristics. Ceramica.

[30] Plonska M., Dzik J. Characterization of Lead Lanthanum Zirconate Titanate Ceramics Co-Doped with Lanthanide Ions. Proceedings of the 7th Forum on New Materials—Part B 2016.

[31] Plonska M., Pisarski W.A., Pisarska J. Luminescence Properties of Ytterbium Activated PLZT Ceramics. Proceedings of the 7th Forum on New Materials—Part B 2016.

[32] De Queiroz T.B., Mohr D., Eckert H., de Camargo A.S.S. (2009). Preparation and structural characterization of rare-earth doped lead lanthanum zirconate titanate ceramics. Solid State Sci..

[33] Bai Y. (2021). Photoresponsive Piezoelectrics. Front. Mater..

[34] Batra V., Kotru S., Varagas M., Ramana C.V. (2015). Optical constants and band gap determination of Pb_0.95_La_0.05_Zr_0.54_Ti_0.46_O_3_ thin films using spectroscopic ellipsometry and UV–visible spectroscopy. Opt. Mater..

[35] Dieke G.H., Crosswhite H.M. (1963). The Spectra of the Doubly and Triply Ionized Rare Earths. Appl. Opt..

[36] Shannigrahi S.R., Tripathy S. (2007). Micro-Raman spectroscopic investigation of rare earth-modified lead zirconate titanate ceramics. Ceram. Int..

[37] Płońska M., Adamczyk M. (2015). Dielectric properties of neodymium-modified PLZT ceramics. Phase Trans..

[38] Sternberg A., Dimza V., Sprogis A., Kapenieks A., Shebanov L., Grinvalds G., Rubulis A., Stumpe R. (1988). Optical and dielectric properties of transparent PLZT ceramics with various defects. Ferroelectrics.

[39] Durruthy-Rodriguez M.D., Gervacio-Arciniega J.J., Hernandez-Garcia M., Yánez-Limon J.M. (2018). Photoluminescence characteristics of soft PZT 53/47 ceramic doped at A and/or B sites. J. Adv. Ceram..

[40] Plonska M., Pisarski W.A. (2016). Excitation and emission of Pr^3+^:PLZT ceramics. Ceram. Int..

[41] Huang C., Xu J., Fang Z., Ai D., Zhou W., Zhao L., Sun J., Wang Q. (2017). Effect of preparation process on properties of PLZT (9/65/35) transparent ceramics. J. Alloys Compd..

[42] Płońska M., Pisarski W.A., Wodecka-Duś B., Czekaj D. (2013). The influence of fabrication conditions on the physical properties of PLZT:Nd^3+^ ceramics. Arch. Metall. Mater..

[43] Patil K.C., Hedgde M.S., Rattan T., Aruna S.T. (2008). Chemistry of Nanocrystalline Oxide Materials, Combustion Synthesis, Properties and Applications.

[44] Patil K.C., Aruna S.T., Mimani T. (2002). Combustion synthesis: An update. Curr. Opin. Solid State Mater. Sci..

[45] Singh G., Tiwari V.S., Tiwari P., Srivasrava A.K., Gupta P.K. (2011). Effect of oxidant-to-fuel ratios on phase formation of PLZT powder; prepared by gel-combustion. J. Alloys Compd..

[46] Bochenek D. (2022). A Combination of Calcination and the spark plasma sintering method in multiferroic ceramic composite technology: Effects of process temperature and dwell time. Materials.

[47] Kröger F., Vink H. (1956). Relations between the Concentrations of Imperfections in Crystalline Solids. Solid State Phys..

[48] Chang T.I., Huang J.L., Lin H.P., Wang S.C., Lu H.H., Wu L., Lin J.F. (2006). Effect of drying temperature on structure, phase transformation of sol-gel derived lead zirconate titanate powders. J. Alloys Compd..

[49] Scott J.F., Dawber M. (2000). Oxygen-vacancy ordering as a fatigue mechanism in perovskite ferroelectrics. Appl. Phys. Lett..

[50] International Centre for Diffraction Data. https://www.icdd.com/?gclid=Cj0KCQjw6J-SBhCrARIsAH0yMZjpLPu8oJKSk86zbcA_VTtlnuyrD17YXNqXCudyEl9Hl9zZI7KhNzYaAtbcEALw_wcB.

[51] Mansour S., Eid A., El-Latif Abd L., Rashad M., Afifi M., Turner J. (2018). Dielectric and piezoelectric performance of gadolinium-doped lead lanthanum zirconate titanate. Int. J. Appl. Ceram. Technol..

[52] Brites C.D.S., Fiaczyk K., Ramalho J.F.C.B., Sójka M., Carlos L.D., Zych E. (2018). Widening the temperature range of luminescent thermometers through the intra- and interconfigurational transitions of Pr^3+^. Adv. Opt. Mater..

[53] Boutinaud P., Mahiou R., Cavalli E., Bettinelli M. (2006). Luminescence properties of Pr^3+^in titanates and vanadates: Towards a criterion to predict ^3^P_0_ emission quenching. Chem. Phys. Lett..

[54] Zou H., Hui X., Wang X., Peng D., Li J., Li Y., Yao X. (2013). Luminescent, dielectric, and ferroelectric properties of Pr doped Bi_7_Ti_4_NbO_21_ multifunctional ceramics. J. Appl. Phys..

[55] Górny A., Sołtys M., Pisarska J., Pisarski W.A. (2019). Germanate glasses co-doped with Ce ^3+^ /Ln ^3+^ (Ln = Pr, Tb, Dy) for white light emitting diodes. Opt. Appl..

[56] https://medium.com/hipster-color-science/a-beginners-guide-to-colorimetry-401f1830b65a.

[57] Tan Q., Viehland D. (1996). Ac-field-dependent structure-property relationships in La-modified lead zirconate titanate: Induced relaxor behavior and domain breakdown in soft ferroelectrics. Phys. Rev. B.

[58] Sato Y., Kanai H., Yamashita Y. (1994). Grain size dependence of dielectric constant for modified (Pb0.63Ba0.37)(Zr0.7Ti0.3)O_3_ ceramic material. Jpn. J. Appl. Phys..

[59] Uchino K., Nomura S. (1982). Critical exponents of the dielectric constants in diffused-phase transition crystals. Ferroelectr. Lett. Sect..

[60] Wang G., Fan Z., Murakami S., Lu Z., Hall D.A., Sinclair D.C., Feteira A., Tan X., Jones J.L., Kleppe A.K. (2019). Origin of the large electrostrain in BiFeO_3_-BaTiO_3_ based lead-free ceramics. J. Mater. Chem. A.

[61] Kim S., Miyauchi R., Sato Y., Nam H., Fujii I., Ueno S., Kuroiwa Y., Wada S. (2023). Piezoelectric Actuation Mechanism Involving Extrinsic Nanodomain Dynamics in Lead-Free Piezoelectrics. Adv. Mater..

[62] Wang X., Lv X., Ma Y., Zhang X., Lyu J., Wu J. (2023). Deciphering the role of A-site ions of AZrO_3_-type dopants in (K, Na)NbO_3_ ceramics. Acta Mater..

[63] Picinin A., Lente M.H., Eiras J.A., Rino J.P. (2004). Theoretical and experimental investigations of polarization switching in ferroelectric materials. Phys. Rev. B.

[64] Bokov A.A., Ye Z.-G. (2006). Recent Progress in Relaxor Ferroelectrics with Perovskite Structure. J. Mater. Sci..

[65] Minhas J.Z., Hasan A.M., Yang Y. (2021). Ferroelectric Materials Based Coupled Nanogenerators. Nanoenergy Adv..

